# Content and Bioaccumulation of Nine Mineral Elements in Ten Mushroom Species of the Genus *Boletus*

**DOI:** 10.1155/2015/165412

**Published:** 2015-06-04

**Authors:** Xue-Mei Wang, Ji Zhang, Tao Li, Yuan-Zhong Wang, Hong-Gao Liu

**Affiliations:** ^1^College of Agronomy and Biotechnology, Yunnan Agricultural University, Kunming 650201, China; ^2^Institute of Medicinal Plants, Yunnan Academy of Agricultural Sciences, Kunming 650200, China; ^3^College of Resources and Environment, Yuxi Normal University, Yuxi 653100, China

## Abstract

Concentrations and bioconcentration potential of nine elements (Ca, Cu, Fe, K, Mg, Mn, Na, P, and Zn) in ten species of wild edible *Boletus* and the corresponding underlying soils were analyzed. The analyses were performed using inductively coupled plasma atomic emission spectrophotometer. *Boletus* showed relative abundant contents of P, K, Fe, Mg, Ca, and Na and less of Zn, Cu, and Mn. Caps compared to stalks were enriched in P, K, Cu, Mg, and Zn, while stalks were enriched in Mn. The elements such as P and K were accumulated (BCF > 1), while Ca, Fe, Mg, Mn, and Na were excluded (BCF < 1) in the fruiting bodies. The correlation analysis indicated high correlations between Cu, Mn, Ca, and Fe in the mushrooms as compared to the corresponding soils. Significant correlations were also obtained between Cu-P (*r* = 0.775), Fe-P (*r* = 0.728), and Zn-P (*r* = 0.76) for caps and Cu-Mg (*r* = 0.721), Fe-Mg (*r* = 0.719), Zn-Mg (*r* = 0.824), and Zn-P (*r* = 0.818) for stalks. The results of this study imply that ability of fungi to accumulate elements from substrate could be influenced by mushroom species and underlying soil substrates.

## 1. Introduction

Mushrooms are traditionally recognized as a valued source of nutrients and a popular delicacy in many countries for their rich contents in proteins, essential minerals, and low energy levels [1]. Edible wild-grown mushrooms consumption has been preferred to cultivated species for their indisputable flavor, texture, and medicinal properties [2, 3]. Proteins of wild edible mushrooms are usually high, commonly ranging between 10% and 60%, which were calculated using the conversion factor of 4.38 [1]. Mushroom gathering has been a very popular leisure and commercial activity in many European and Asian countries [4].

Macromycetes in nature play a major role in biogeochemical transformation of macro- and microelements essential to humans [5]. Wild mushrooms can absorb large amounts of water and minerals within a relatively short period due to large area of mycelium overgrowing the surface layer of soil [6]. Abundance of minerals such as P, K, Ca, Mg, Na, Fe, Mn, Zn, and Cu desired in human nutrition has been reported in edible wild-grown mushrooms; meanwhile, undesired high levels of toxic elements including As, Hg, Cd, and Pb were also found in previous studies [5]. Bioconcentration factor (BCF) is the ratio of the element content in fruiting body (cap or stalk) to the content in underlying substrate, which can express the ability of fungi to accumulate elements from substrate [7]. If BCF exceeds unity (BCF > 1) it indicates the accumulation of elements in fungus, and if BCF is below unity (BCF < 1) it excludes bioaccumulation [8]. Bioaccumulation capacity of mushroom is usually affected by fungal lifestyle, age of fruiting body, specific species and element, and environment such as pH, organic matter, and pollution [5].

Rich resources of wild edible fungi are distributed in Yunnan province (southwest of China) due to the mild and rainy climate. Laterite, lateritic red loam, red soil, yellow soil, and brown soil are the main agrotypes in Yunnan [9]. Contents of elements such as Cu, Fe, Pb, Cd, Co, Se, Ni, V, Tl, Sc, and Ti in soil are rather high, which would be the primary sources of elements to fungi [10]. More than 880 species of wild-grown mushrooms were identified as edible in Yunnan province, which account for 80% in China and 40% in the world [11]. Export volume of wild edible mushrooms in Yunnan was 105000 and 135000 tons, and the exports were 1.21 and 1.43 million dollars in 2010 and 2011, respectively. Species of the genus* Boletus* and* Tricholoma matsutake* are the main export varieties, which account for 90% of the total export in Yunnan [12].

Species of the genus* Boletus* are a popular food throughout the world because of their particular aroma, sensory qualities, texture, and biologically active compounds. Flavor of the favorite dried species such as* Boletus edulis*,* Boletus aereus*, and* Boletus badius* includes odor and marvelous taste of nuttiness, earthiness, and meatiness [13]. 199 species of Boletales were identified as edible in China, among which 144 species were distributed in Yunnan province [14].* Boletus* is abundant in proteins, carbohydrates, vitamins, and essential elements required in the human body. Furthermore, its history of traditional use in oriental therapies and modern clinical practice has been reported such as use as for its anti-inflammatory, antimicrobial, antioxidant, antiviral, antitumor, and immunomodulatory activities and its use for reduction of blood glucose levels [15]. However, species such as* Boletus edulis*,* Boletus badius*,* Boletus griseus*, and* Boletus pinophilus* contained high contents of toxic Cd, Hg, As, and Pb [5, 16, 17]. Also undesired Ag and Sb can be elevated in some wild-grown mushrooms [18, 19].

There was little information available about bioaccumulation ability of elements in* Boletus* family in China. Since these mushrooms have been traditionally used for consumption by local people and trading, the aim of this study was to determine the contents of nine elements (Ca, Cu, Fe, K, Mg, Mn, Na, P, and Zn) in ten species of edible* Boletus* and the underlying soil to investigate the relationship between fruiting body and habitat.

## 2. Materials and Methods

### 2.1. Sampling

Mature fruiting bodies of ten edible species of the genus* Boletus* were collected from Yunnan province (southwest of China) during summer and autumn in 2012. The species, habitat, and number of fruiting bodies are given in Table 1. The underlying surface layers (0 to 10 cm) were collected after removing the superficial layer of litter and organic detritus.

Fresh fruiting bodies of* Boletus* were cleaned from plant and substrate debris with a plastic knife and divided into two parts (caps and stalk) and then washed with deionized water. Each sample was dried in an electrically heated oven at 65°C to constant weight. Dried samples were pulverized with laboratory mill and kept in sealed polyethylene bags in dry conditions. Substrate samples were air-dried at room temperature for several weeks and passed through a 100-mesh sieve.

### 2.2. Digestion Process

Dried mushroom samples were weighed as 0.3 g accurately and placed into the polytetrafluoroethylene (PTFE) pressure vessels. 4 mL HNO_3_ (65%) and 2 mL H_2_O_2_ (30%) were added to the vessel and digested under pressure in an automatic microwave digestion system, Ethos One (Milestone, Italy). The extract was further diluted to 25 mL using deionized water.

Soil samples (0.1 g) were placed into the polytetrafluoroethylene beaker and digested and mixed with 6 mL HNO_3_ (65%) and 1 mL HClO_4_ (72%) on an electric hot plate at 170°C, removing and cooling the samples when they turned into paste. Then the paste was dissolved in 10 mL HF (40%) and 0.5 mL HClO_4_ (72%) and heated again at 210~220°C until the acid evaporated completely. Further, 10 mL HCl (38%) was added and heated until dissolved and diluted with distilled water up to 25 mL.

### 2.3. Analytical Determination

All elements (Ca, Cu, Fe, K, Mg, Mn, Na, P, and Zn) contents in dry weight (dw) of mushrooms and soils samples were determined using inductively coupled plasma atomic emission spectrophotometer (ICPE-9000, Shimadzu, Japan). The repeatability for all determined elements was less than 5%. Limits of quantitation (LOQ) for Ca, Cu, Fe, K, Mg, Mn, Na, P, and Zn were 0.04, 0.17, 0.18, 2.16, 0.04, 0.01, 1.53, 0.44, and 0.05 mg/kg dw, respectively. The efficiency of the process was validated with 94–107% recovery success using certified reference materials, namely, tea leaves (GBW07605) and soils (GBW07406), both produced by the Institute of Geophysical and Geochemical Exploration in Beijing, China.

## 3. Results and Discussion

### 3.1. Element Concentrations

The element contents in fruiting bodies of* Boletus* (cap and stalk) and underlying soil samples are presented in Table 2. As previously reported, fruiting bodies of* Boletus* can be considered as rich in P, K, Na, Ca, and Mg and also abundant in trace elements such as Cu, Zn, and Mn. Among the minerals determined in this study, K, P, Fe, Mg, and Ca exhibited higher concentrations than Na, Zn, Cu, and Mn, which is in accordance with the literature [7]. While* Boletus* collected from Yunnan contained lower levels of K, Na, Ca, Mg, Fe, and Mn than other reports [20], significant differences were obtained not only among various* Boletus* species but also within different part of fruiting body (Table 2). Similarly to previous studies, the ranges of concentrations spread over one to two orders of magnitude often occurred [21, 22].

Among the mushroom species analyzed, the greatest contents of Cu, K, Mg, Na, and P were quantified in the caps and stalks of* Boletus pallidus*, and Mn and Zn were found in* Boletus luridus* and* Boletus rubellus*. Highest Ca and Fe contents were found in the caps of* Boletus brunneissimus* and stipes of* Boletus edulis*, namely, 669 and 4200 mg/kg dw, respectively. In the study by Zhou and Yin (2008) [23],* Boletus edulis* showed higher contents of K, Na, Ca, Mg, and Fe than* Boletus speciosus* and* Boletus aereus*. In our early study, values of 13.6, 121, 248, and 20.3 mg/kg dw for Cu, Fe, Mg, and Zn were determined in* Boletus tomentipes*, respectively, which are lower than the present results [24]. Similarly, contents of K, Ca, Fe, Mg, and Zn for* Boletus speciosus* and* Boletus griseus* collected from Yunnan province in 2009 were lower than the data in Table 2 [25].

K contents in caps and stalks of* Boletus edulis* (cap: 29000 mg/kg, stalk: 20000 mg/kg dw) and* Boletus subtomentosus* (cap: 46000 mg/kg, stalk: 43000 mg/kg dw) from Poland were much higher than the values in our study (cap: 9017–18968 mg/kg, stalk: 4865–15325 mg/kg dw) [8, 26]. P contents were found at the ranges of 4942–10611 mg/kg dw and 2176–5621 mg/kg dw for caps and stalks of* Boletus*, respectively. Higher values were found to be 8700–16000 mg/kg dw in caps and 4100–7500 mg/kg dw in stalks of* Macrolepiota procera*, and lower values of 640–4490 mg/kg dw were for the fruiting body of thirty macrofungi species from Turkey [27, 28].

The range of Fe contents in this study was 376–1600 in caps and 357–4200 mg/kg dw in stalks, which are much higher than the values previously reported [8, 29]. Similar values were found to be 150–1741 mg/kg dw, and a particularly higher value was 11460 mg/kg dw for* Lepista nuda* [30, 31]. Reported Ca contents in* Boletus edulis*,* Boletus speciosus*, and* Boletus tomentipes* were 913.9, 1147.6, and 1097.4 mg/kg dw, which are higher than the values observed in this study [32].

Mg and Zn concentrations in different* Boletus* were in the ranges of 386–1239 and 45–223 mg/kg dw for caps and 279–935 and 33–131 mg/kg dw for stalks, respectively, which coincide with the literature reports [5]. Cu and Mn contents in* Boletus badius*,* Boletus luridus*, and* Boletus edulis* were found to be lower than the results in our study, that is, 33–78 and 17–67 mg/kg dw for caps and 16–39 and 26–85 mg/kg dw for stalks, respectively [5, 33, 34]. Other species such as* Macrolepiota procera* and* Lepista nuda* contained higher content of Cu (236.5 mg/kg dw) and Mn (480 mg/kg dw) [6, 31]. The range of Na contents in caps was 105–601 mg/kg dw and 90–500 mg/kg dw in stalks, which are lower than values in species such as* Boletus subtomentosus* (77–1200 mg/kg dw),* Pleurotus ostreatus* (220–1400 mg/kg dw), and* Agaricus bisporus* (16000–25000 mg/kg dw) [26, 35, 36].

Uneven distribution of elements between caps and stalks is shown in Table 2. The caps of* Boletus* contained higher contents of K, P, Cu, Mg, and Zn and lower contents of Mn when compared to stalks. Reports also suggested similar results for these elements in* Boletus edulis*,* Boletus subtomentosus*, and* Boletus scaber* [26, 37, 38]. However, the contents of Ca, Fe, and Na between caps and stalks have no certain regularity (Table 2). Contents of Cu, P, and Zn in caps are approximately twice or even thrice higher than in stalks, and similar findings were obtained in the review of Falandysz and Borovička [5]. As can be seen from Table 2, both caps and stalks can be considered as abundant in P, K, Fe, Mg, Zn, and Ca, and* Boletus* could be considered potential dietary sources of these essential elements to humans.

According to the EU Scientific Committee, 60 kg of body weight was used for intake calculations. For intake calculations, usually a 300 g portion of fresh mushrooms per meal is assumed, which contains 30 g of dry matter [39]. The elements intakes by a normal (60 kg) consumer in mg* per* serving for all studied fruiting bodies are presented in Table 3.

### 3.2. Bioconcentration Factor (BCF)

Bioconcentration factor (BCF) values in caps and stalks of* Boletus* are summarized in Table 4. Among the elements determined, P and K exhibit BCF > 1, except for BCF for stalk of* Boletus edulis* (Table 4), while Ca, Fe, Mg, Mn, and Na are bioexcluded (BCF < 1) by the* Boletus* species studied. Although a certain element in the soil is abundant and could accumulate to relatively high contents in the mushroom, BCF below 1 may be obtained, which was reported for Ca, Fe, Mg, Mn, and Na in a recent study [8].

Wild mushrooms such as* Pholiota nameko*,* Tricholoma quercicol*, and* Ramaria flava* collected from Sichuan province (China) showed lower BCF values for potassium, that is, 0.84, 0.48, and 0.5, respectively [40]. Higher BCF values for K, Na, Zn, Cu, P, and Mg were found in* Boletus subtomentosus*, namely, 4400, 340, 110, 77, 40, and 21 for caps and 3900, 310, 67, 27, 18, and 11 for stalks, respectively [26].* Boletus edulis* collected from Poland also showed higher levels of BCF values for Cu and Zn, that is, 3.6–31 for caps and 1.7–16 for stipes [41]. In the study by Ma et al., BCF values of Zn and Cu were less than 0.01 in five kinds of wild edible* Boletus* from China [42]. In early studies, the blockage of Ca, Fe, and Mn (BCF < 1) was reported in fruiting body of* Macrolepiota procera*,* Leccinum griseum*, and* Tricholoma quercicol* [27, 29, 40, 43], which agrees with our results.

### 3.3. Correlation Analysis

Matrices of correlation coefficients among elements concentrations of the fruiting body (caps and stalks) and underlying soils are presented in Tables 5 and 6. Cu (*r* = 0.556) and Mn (*r* = 0.431) had significant correlations in caps, and high correlation were seen between Ca (*r* = 0.727), Cu (*r* = 0.6), and Fe (*r* = 0.569) in stalks. Similar results were obtained for Cu (*r* = 0.478), Mn (*r* = 0.389), and Fe (*r* = 0.417) in* Verpa conica*,* Tricholoma terreum*, and* Boletus badius*, respectively [44]. Meanwhile, significant correlations were also found between Ca-Fe (*r* = 0.681), Cu-P (*r* = 0.775), Fe-P (*r* = 0.728), Mn-P (*r* = 0.667), Zn-K (*r* = 0.673), Zn-Mg (*r* = 0.675), and Zn-P (*r* = 0.76) for caps. For stalks, significant correlations were found between Cu-Mg (*r* = 0.721), Cu-P (*r* = 0.659), Fe-Mg (*r* = 0.719), Fe-P (*r* = 0.693), Zn-Mg (*r* = 0.824), Zn-P (*r* = 0.684), and Zn-P (*r* = 0.818).

## 4. Conclusion

The present study gives an overview for the levels of elements accumulation, determined in caps and stalks of ten species of* Boletus* from Yunnan province, southwest of China. From the nutritional point of view, these* Boletus* could be considered as a potential dietary source of essential elements such as P, K, Mg, Cu, Zn, Mn, and Fe. Elements K, P, Cu, Mg, and Zn are preferably accumulated in the cap of* Boletus*, while Mn is preferably accumulated in stalk. Depending on the environmental conditions and mushroom species, the values of BCF varied highly depending on chemical element and were >1 for P and K, while they were <1 for Ca, Fe, Mg, Mn, and Na. The correlation analysis showed that Cu, Mn, Ca, and Fe contents were highly correlated in the mushrooms as compared to the corresponding soils. Significant correlations were also obtained between Cu-P (*r* = 0.775), Fe-P (*r* = 0.728), and Zn-P (*r* = 0.76) for caps and Cu-Mg (*r* = 0.721), Fe-Mg (*r* = 0.719), Zn-Mg (*r* = 0.824), and Zn-P (*r* = 0.818) for stalks.

## Figures and Tables

**Table 1:** Families, habitat, and number of analyzed fruiting bodies.

Species	Habitat	Number
*Boletus speciosus* Frost	On mixed woods ground	10
*Boletus griseus *Frost	On needles, oak, and chestnut forest ground	10
*Boletus luridus *Schaeff.	On broadleaf or mixed woods ground	8
*Boletus umbriniporus* Hongo	On forest ground	8
*Boletus tomentipes* Earle	On needles and broadleaf forest ground	7
*Boletus edulis* Bull.	On mixed coniferous and broadleaf forest ground	10
*Boletus pallidus* Frost	On shaw ground	9
*Boletus brunneissimus* W.F. Chiu	On mixed pine tree and katue forest ground	9
*Boletus rubellus *Krombh.	On broadleaf or mixed woods ground	7
*Boletus aereus *Bull.	On oak forest ground	7

**Table 2:** Elements content in the caps and stipes of *Boletus* and underlying soil (mg/kg dw, mean, SD).

Species		Ca	Cu	Fe	K	Mg	Mn	Na	P	Zn
*Boletus speciosus *	Cap	172 ± 36	66 ± 12	1021 ± 216	9017 ± 453	909 ± 158	21 ± 6	130 ± 76	5612 ± 587	148 ± 25
	Stipe	141 ± 91	22 ± 5	959 ± 232	6105 ± 829	504 ± 307	30 ± 2	118 ± 13	2218 ± 573	56 ± 26
	Soil	449 ± 234	35 ± 21	20081 ± 3426	3169 ± 432	1134 ± 673	140 ± 32	876 ± 27	656 ± 32	63 ± 2

*Boletus griseus *	Cap	538 ± 56	40 ± 13	1166 ± 133	17310 ± 667	577 ± 54	48 ± 22	324 ± 26	7126 ± 334	205 ± 55
	Stipe	512 ± 32	24 ± 6	1194 ± 244	15308 ± 565	407 ± 75	63 ± 22	226 ± 13	4789 ± 454	125 ± 52
	Soil	1076 ± 32	39 ± 13	26989 ± 2411	5373 ± 3458	1169 ± 113	459 ± 89	1020 ± 675	1686 ± 242	113 ± 7

*Boletus luridus *	Cap	475 ± 29	65 ± 13	1600 ± 345	11206 ± 677	556 ± 81	67 ± 23	105 ± 12	6832 ± 565	131 ± 18
	Stipe	395 ± 165	20 ± 12	1117 ± 212	7594 ± 434	340 ± 223	85 ± 21	90 ± 27	2742 ± 345	51 ± 23
	Soil	2438 ± 341	54 ± 23	28210 ± 788	6665 ± 178	2694 ± 252	773 ± 95	2292 ± 321	1293 ± 132	87 ± 54

*Boletus umbriniporus *	Cap	166 ± 33	43 ± 11	747 ± 256	10740 ± 234	431 ± 56	21 ± 4	421 ± 67	5325 ± 453	90 ± 23
	Stipe	95 ± 12	34 ± 13	357 ± 32	8360 ± 568	279 ± 32	26 ± 5	451 ± 134	2176 ± 234	33 ± 6
	Soil	758 ± 54	26 ± 12	19966 ± 4663	5154 ± 126	990 ± 645	315 ± 13	2071 ± 234	1778 ± 543	110 ± 78

*Boletus tomentipes *	Cap	99 ± 45	48 ± 6	376 ± 32	11488 ± 345	386 ± 234	17 ± 2	170 ± 11	5155 ± 324	115 ± 45
	Stipe	113 ± 34	28 ± 5	751 ± 65	10775 ± 454	314 ± 32	38 ± 3	107 ± 76	3089 ± 235	90 ± 6
	Soil	1081 ± 38	53 ± 6	38286 ± 479	5761 ± 46	1118 ± 67	281 ± 37	681 ± 89	850 ± 46	108 ± 23

*Boletus edulis *	Cap	320 ± 26	51 ± 34	931 ± 82	9413 ± 567	429 ± 54	26 ± 11	191 ± 53	6615 ± 343	88 ± 4
	Stipe	317 ± 33	32 ± 4	4200 ± 678	4865 ± 245	297 ± 65	70 ± 13	122 ± 23	2429 ± 545	41 ± 33
	Soil	1624 ± 45	152 ± 20	52613 ± 656	8533 ± 234	3665 ± 867	945 ± 77	1972 ± 45	1495 ± 22	118 ± 67

*Boletus pallidus *	Cap	342 ± 234	78 ± 6	1475 ± 56	18968 ± 676	1239 ± 82	36 ± 2	601 ± 4	10611 ± 234	45 ± 76
	Stipe	378 ± 56	39 ± 4	1823 ± 341	15325 ± 459	935 ± 240	39 ± 22	500 ± 345	5621 ± 248	37 ± 9
	Soil	2624 ± 787	261 ± 55	83294 ± 676	3617 ± 89	1745 ± 456	1092 ± 88	1288 ± 67	1330 ± 234	396 ± 78

*Boletus brunneissimus *	Cap	669 ± 34	33 ± 23	741 ± 78	11757 ± 346	759 ± 21	29 ± 8	472 ± 44	4942 ± 986	167 ± 50
	Stipe	154 ± 78	16 ± 4	702 ± 23	10320 ± 654	513 ± 78	38 ± 2	216 ± 56	2949 ± 873	88 ± 57
	Soil	1879 ± 245	50 ± 14	32165 ± 343	6765 ± 652	1307 ± 45	744 ± 22	891 ± 454	2007 ± 13	145 ± 29

*Boletus rubellus *	Cap	285 ± 67	56 ± 34	1376 ± 46	11606 ± 428	773 ± 65	41 ± 16	203 ± 46	5888 ± 106	223 ± 54
	Stipe	632 ± 35	35 ± 21	3488 ± 785	10732 ± 345	631 ± 76	71 ± 13	340 ± 78	4853 ± 906	131 ± 86
	Soil	3289 ± 442	159 ± 45	57870 ± 466	5675 ± 545	1802 ± 42	682 ± 24	825 ± 74	2176 ± 346	251 ± 57

*Boletus aereus *	Cap	140 ± 21	61 ± 41	654 ± 45	11365 ± 343	758 ± 67	26 ± 3	546 ± 50	5467 ± 535	165 ± 24
	Stipe	139 ± 6	37 ± 5	2323 ± 210	7905 ± 676	556 ± 35	77 ± 4	291 ± 65	2497 ± 342	75 ± 6
	Soil	687 ± 43	60 ± 3	34440 ± 652	5675 ± 874	1535 ± 257	378 ± 35	2393 ± 256	695 ± 57	77 ± 20

**Table 3:** Daily metal intakes by a normal, 60 kg consumer in mg/serving.

Species		Ca	Cu	Fe	K	Mg	Mn	Na	P	Zn
*Boletus speciosus *	Cap	5.16 ± 1.08	1.98 ± 0.36	30.6 ± 6.48	271 ± 13.6	27.3 ± 4.74	0.63 ± 0.18	3.9 ± 2.28	168.4 ± 17.6	4.44 ± 0.75
	Stipe	4.23 ± 2.73	0.66 ± 0.15	28.8 ± 6.96	183 ± 24.9	15.1 ± 9.21	0.9 ± 0.06	3.54 ± 0.39	66.5 ± 17.3	1.68 ± 0.78

*Boletus griseus *	Cap	16.1 ± 1.68	1.2 ± 0.39	35.0 ± 3.99	519 ± 20.0	17.3 ± 1.62	1.44 ± 0.66	9.72 ± 0.78	214 ± 10.0	6.15 ± 1.65
	Stipe	15.4 ± 0.96	0.72 ± 0.18	35.8 ± 7.32	459 ± 17.0	12.2 ± 2.25	1.89 ± 0.66	6.78 ± 0.39	144 ± 13.6	3.75 ± 1.56

*Boletus luridus *	Cap	14.3 ± 0.87	1.95 ± 0.39	48 ± 10.4	336 ± 20.3	16.7 ± 21.43	2.01 ± 0.69	3.15 ± 0.36	205 ± 17.0	3.93 ± 0.54
	Stipe	11.9 ± 4.95	0.6 ± 0.36	33.5 ± 6.36	228 ± 13.0	10.2 ± 6.69	2.55 ± 0.63	2.7 ± 0.81	82.3 ± 10.4	1.53 ± 0.69

*Boletus umbriniporus *	Cap	4.98 ± 0.99	1.29 ± 0.33	22.4 ± 7.68	322 ± 7.02	12.9 ± 1.68	0.63 ± 0.12	12.6 ± 2.01	160 ± 13.4	2.7 ± 0.69
	Stipe	2.85 ± 0.36	1.02 ± 0.39	10.7 ± 0.96	251 ± 17.0	8.37 ± 0.96	0.78 ± 0.15	13.5 ± 4.02	65.3 ± 7.02	0.99 ± 0.18

*Boletus tomentipes *	Cap	2.97 ± 1.35	1.44 ± 0.18	11.3 ± 0.96	345 ± 10.4	11.6 ± 7.02	0.51 ± 0.06	5.1 ± 0.33	155 ± 9.72	3.45 ± 1.35
	Stipe	3.39 ± 1.02	0.84 ± 0.15	22.5 ± 1.95	323 ± 13.6	9.42 ± 0.96	1.14 ± 0.09	3.21 ± 2.28	92.7 ± 7.05	2.7 ± 0.18

*Boletus edulis *	Cap	9.6 ± 0.78	1.53 ± 1.02	27.9 ± 2.46	282 ± 17.0	12.9 ± 1.62	0.78 ± 0.33	5.73 ± 1.59	198 ± 10.3	2.64 ± 0.12
	Stipe	9.5 ± 0.99	0.96 ± 0.12	126 ± 20.3	146 ± 7.35	8.91 ± 1.95	2.1 ± 0.39	3.66 ± 0.69	72.9 ± 16.4	1.23 ± 0.99

*Boletus pallidus *	Cap	10.3 ± 7.02	2.34 ± 0.18	44.3 ± 1.68	569 ± 20.3	37.2 ± 2.46	1.08 ± 0.06	18.0 ± 0.12	318 ± 7.02	1.35 ± 2.28
	Stipe	11.3 ± 1.68	1.17 ± 0.12	54.7 ± 10.2	460 ± 13.8	28.1 ± 7.2	1.17 ± 0.66	15 ± 10.4	168.6 ± 7.44	1.11 ± 0.27

*Boletus brunneissimus *	Cap	20.1 ± 1.02	0.99 ± 0.69	22.2 ± 2.34	353 ± 10.4	22.8 ± 0.63	0.87 ± 0.24	14.2 ± 1.32	148 ± 29.6	5.01 ± 1.5
	Stipe	4.62 ± 2.34	0.48 ± 0.12	21.1 ± 0.69	310 ± 19.6	15.4 ± 2.34	1.14 ± 0.06	6.48 ± 1.68	88.5 ± 26.2	2.64 ± 1.71

*Boletus rubellus *	Cap	8.55 ± 2.01	1.68 ± 1.02	41.3 ± 1.38	348 ± 12.8	23.2 ± 1.95	1.23 ± 0.48	6.09 ± 1.38	177 ± 3.18	6.69 ± 1.62
	Stipe	19.0 ± 1.05	1.05 ± 0.63	105 ± 23.6	322 ± 10.4	18.9 ± 2.28	2.13 ± 0.39	10.2 ± 2.34	146 ± 27.2	3.93 ± 2.58

*Boletus aereus *	Cap	4.2 ± 0.63	1.83 ± 1.23	19.6 ± 1.35	341 ± 10.3	22.7 ± 2.01	0.78 ± 0.09	16.4 ± 1.5	164 ± 16.1	4.95 ± 0.72
	Stipe	4.17 ± 0.18	1.11 ± 0.15	69.7 ± 6.3	237 ± 20.3	16.7 ± 1.05	2.31 ± 0.12	8.73 ± 1.95	74.9 ± 10.3	2.25 ± 0.18

**Table 4:** Bioconcentration factor (BCF) values of caps and stipes of *Boletus*.

Species		Ca	Cu	Fe	K	Mg	Mn	Na	P	Zn
*Boletus speciosus *	Caps	0.38 ± 0.1	1.89 ± 0.24	0.05 ± 0.01	2.85 ± 0.57	0.80 ± 0.03	0.15 ± 0.02	0.15 ± 0.04	8.55 ± 1.27	2.35 ± 0.78
	Stipes	0.31 ± 0.12	0.63 ± 0.29	0.05 ± 0.04	1.93 ± 0.75	0.44 ± 0.18	0.21 ± 0.08	0.13 ± 0.02	3.38 ± 1.69	0.89 ± 0.23

*Boletus griseus *	Caps	0.5 ± 0.08	1.03 ± 0.3	0.04 ± 0.01	3.22 ± 0.59	0.49 ± 0.07	0.1 ± 0.04	0.32 ± 0.03	4.23 ± 0.88	1.81 ± 0.12
	Stipes	0.48 ± 0.05	0.62 ± 0.06	0.04 ± 0.01	2.85 ± 1.32	0.35 ± 0.03	0.14 ± 0.02	0.22 ± 0.05	2.84 ± 0.39	1.11 ± 0.45

*Boletus luridus *	Caps	0.2 ± 0.04	1.2 ± 0.37	0.06 ± 0.05	1.68 ± 0.67	0.21 ± 0.11	0.1 ± 0.03	0.05 ± 0.02	5.28 ± 1.84	1.51 ± 0.34
	Stipes	0.16 ± 0.08	0.37 ± 0.02	0.04 ± 0.01	1.14 ± 0.52	0.13 ± 0.01	0.11 ± 0.05	0.04 ± 0.01	2.12 ± 1.55	0.59 ± 0.33

*Boletus umbriniporus *	Caps	0.2 ± 0.13	1.7 ± 0.76	0.04 ± 0.03	2.08 ± 1.27	0.44 ± 0.26	0.1 ± 0.08	0.2 ± 0.12	2.99 ± 0.65	0.82 ± 0.37
	Stipes	0.13 ± 0.03	1.31 ± 0.54	0.02 ± 0.01	1.62 ± 0.75	0.28 ± 0.02	0.08 ± 0.04	0.22 ± 0.16	1.22 ± 0.48	0.3 ± 0.19

*Boletus tomentipes *	Caps	0.1 ± 0.05	0.9 ± 0.53	0.01 ± 0.04	1.99 ± 0.45	0.35 ± 0.21	0.1 ± 0.02	0.2 ± 0.08	6.06 ± 2.14	1.06 ± 0.65
	Stipes	0.1 ± 0.04	0.53 ± 0.3	0.02 ± 0.03	1.87 ± 0.56	0.28 ± 0.03	0.14 ± 0.01	0.16 ± 0.09	3.63 ± 0.73	0.8 ± 0.21

*Boletus edulis *	Caps	0.2 ± 0.04	0.3 ± 0.17	0.02 ± 0.01	1.1 ± 0.93	0.12 ± 0.04	0.03 ± 0.01	0.1 ± 0.07	4.42 ± 1.3	0.75 ± 0.23
	Stipes	0.2 ± 0.06	0.21 ± 0.03	0.08 ± 0.07	0.57 ± 0.01	0.08 ± 0.02	0.07 ± 0.03	0.06 ± 0.02	1.62 ± 0.43	0.3 ± 0.11

*Boletus pallidus *	Caps	0.1 ± 0.06	0.3 ± 0.14	0.02 ± 0.01	5.2 ± 1.46	0.71 ± 0.15	0.03 ± 0.02	0.5 ± 0.27	7.98 ± 2.19	0.11 ± 0.04
	Stipes	0.1 ± 0.04	0.15 ± 0.02	0.02 ± 0.01	4.24 ± 0.58	0.54 ± 0.09	0.04 ± 0.03	0.39 ± 0.14	4.23 ± 0.88	0.1 ± 0.02

*Boletus brunneissimus *	Caps	0.36 ± 0.07	0.66 ± 0.28	0.02 ± 0.01	1.74 ± 0.42	0.58 ± 0.01	0.04 ± 0.02	0.53 ± 0.23	2.46 ± 0.67	1.15 ± 0.17
	Stipes	0.08 ± 0.04	0.32 ± 0.08	0.02 ± 0.01	1.53 ± 1.2	0.39 ± 0.14	0.05 ± 0.01	0.24 ± 0.16	1.47 ± 0.28	0.61 ± 0.03

*Boletus rubellus *	Caps	0.09 ± 0.02	0.35 ± 0.16	0.02 ± 0.01	2.05 ± 1.07	0.43 ± 0.29	0.06 ± 0.02	0.25 ± 0.04	2.71 ± 0.78	0.89 ± 0.06
	Stipes	0.19 ± 0.07	0.22 ± 0.1	0.06 ± 0.01	1.89 ± 0.76	0.35 ± 0.2	0.1 ± 0.06	0.41 ± 0.27	2.23 ± 0.55	0.52 ± 0.12

*Boletus aereus *	Caps	0.2 ± 0.09	1.02 ± 0.34	0.02 ± 0.01	2.0 ± 1.07	0.49 ± 0.23	0.07 ± 0.02	0.23 ± 0.01	7.87 ± 2.09	2.14 ± 0.24
	Stipes	0.2 ± 0.04	0.62 ± 0.01	0.07 ± 0.03	1.39 ± 0.56	0.36 ± 0.21	0.2 ± 0.17	0.12 ± 0.04	3.59 ± 1.22	0.97 ± 0.43

**Table 5:** Correlation matrix that gives the relationship between element concentrations of caps and underlying soil.

Elements in soils	Elements in mushrooms								
	Ca	Cu	Fe	K	Mg	Mn	Na	P	Zn
Ca	0.406	0.262	0.681^*∗*^	0.299	0.322	0.57	−0.031	0.445	0.065
Cu	0.021	0.556	0.487	0.475	0.58	0.1	0.289	0.775^*∗∗*^	−0.392
Fe	0.01	0.492	0.382	0.509	0.54	0.061	0.322	0.728^*∗*^	−0.355
K	0.327	−0.477	−0.162	−0.362	−0.644^*∗*^	0.159	−0.233	−0.305	0.067
Mg	0.173	0.242	0.372	−0.217	−0.176	0.345	−0.318	0.257	−0.262
Mn	0.52	0.253	0.563	0.403	0.328	0.431	0.241	0.667^*∗*^	−0.336
Na	−0.131	0.214	0.09	−0.217	−0.232	0.22	0.174	0.046	−0.321
P	0.582	−0.5	0.292	0.186	−0.092	0.294	0.111	0.016	0.276
Zn	0.137	0.423	0.483	0.673^*∗*^	0.675^*∗*^	0.128	0.456	0.760^*∗*^	−0.295

^*∗*^Correlation is significant at the 0.05 level (2-tailed).

^*∗∗*^Correlation is significant at the 0.01 level (2-tailed).

**Table 6:** Correlation matrix that gives the relationship between element concentrations of stipes and underlying soil.

Elements in soils	Elements in mushrooms								
	Ca	Cu	Fe	K	Mg	Mn	Na	P	Zn
Ca	0.727^*∗*^	0.133	0.423	0.307	0.469	0.374	0.234	0.62	0.204
Cu	0.482	0.6	0.6	0.33	0.721^*∗*^	0.112	0.472	0.659^*∗*^	−0.156
Fe	0.452	0.601	0.569	0.405	0.719^*∗*^	0.113	0.452	0.693^*∗*^	−0.057
K	0.094	−0.173	0.445	−0.384	−0.544	0.537	−0.422	−0.307	0.054
Mg	0.336	0.091	0.718^*∗*^	−0.439	−0.145	0.628	−0.316	−0.103	−0.318
Mn	0.495	0.181	0.497	0.224	0.435	0.334	0.233	0.474	−0.197
Na	−0.164	0.281	0.175	−0.464	−0.253	0.47	0.089	−0.442	−0.571
P	0.522	−0.056	0.171	0.295	0.027	0.05	0.359	0.378	0.338
Zn	0.466	0.505	0.26	0.626	0.824^*∗∗*^	−0.126	0.684^*∗*^	0.818^*∗∗*^	−0.012

^*∗*^Correlation is significant at the 0.05 level (2-tailed).

^*∗∗*^Correlation is significant at the 0.01 level (2-tailed).

## References

[1] Wang X.-M., Zhang J., Wu L.-H. (2014). A mini-review of chemical composition and nutritional value of edible wild-grown mushroom from China. *Food Chemistry*.

[2] Zeng X., Suwandi J., Fuller J., Doronila A., Ng K. (2012). Antioxidant capacity and mineral contents of edible wild Australian mushrooms. *Food Science and Technology International*.

[3] Vamanu E., Nita S. (2013). Antioxidant capacity and the correlation with major phenolic compounds, anthocyanin, and tocopherol content in various extracts from the wild edible *Boletus edulis* mushroom. *BioMed Research International*.

[4] Kalač P. (2013). A review of chemical composition and nutritional value of wild-growing and cultivated mushrooms. *Journal of the Science of Food and Agriculture*.

[5] Falandysz J., Borovička J. (2013). Macro and trace mineral constituents and radionuclides in mushrooms—health benefits and risks. *Applied Microbiology and Biotechnology*.

[6] Mazurkiewicz N., Podlasińska J. (2014). Bioaccumulation of trace elements in wild-growing edible mushrooms from Lubuskie voivodeship, Poland. *Chemistry and Ecology*.

[7] Kalač P. (2010). Trace element contents in European species of wild growing edible mushrooms: a review for the period 2000–2009. *Food Chemistry*.

[8] Frankowska A., Ziołkowska J., Bielawski L., Falandysz J. (2010). Profile and bioconcentration of minerals by King Bolete (*Boletus edulis*) from the Płocka Dale in Poland. *Food Additives and Contaminants: Part B Surveillance*.

[9] Yu G. F., Chen Y. S. (1998). Soil distribution in Yunnan. *Journal of Yunnan University*.

[10] Zeng Z. H., Zeng X. P. (2003). The relationship between cancer death rate and chemical elements in soil of Yunnan province. *Yunnan Geology*.

[11] Wu S.-R., Luo X.-L., Liu B., Gui M.-Y. (2010). Analyse and advise to research and development of wild edible fungi. *Food Science and Technology*.

[12] Zhang Q., Yang X. F. (2013). Export status, problems and countermeasures of wild mushrooms in Yunnan province. *Technology and Market*.

[13] Jaworska G., Bernaś E. (2009). The effect of preliminary processing and period of storage on the quality of frozen *Boletus edulis* (Bull: Fr.) mushrooms. *Food Chemistry*.

[14] Tai L. M., Zhao C. Y., Guo X., Ma M., Liu B. (2013). Prospects of exploitation and utilization on several kinds of high economic value of wild edible and medicinal fungi resource in Yunnan. *Edible Fungi of China*.

[15] Heleno S. A., Barros L., Sousa M. J., Martins A., Santos-Buelga C., Ferreira I. C. F. R. (2011). Targeted metabolites analysis in wild *Boletus* species. *LWT—Food Science and Technology*.

[16] Cocchi L., Vescovi L., Petrini L. E., Petrini O. (2006). Heavy metals in edible mushrooms in Italy. *Food Chemistry*.

[17] Zhang D., Gao T., Ma P., Luo Y., Su P. (2008). Bioaccumulation of heavy metal in wild growing mushrooms from Liangshan Yi Nationality Autonomous Prefecture, China. *Wuhan University Journal of Natural Sciences*.

[18] Falandysz J., Bona H., Danisiewicz D. (1994). Silver content of wild-grown mushrooms from Northern Poland. *Zeitschrift für Lebensmittel-Untersuchung und Forschung*.

[19] Borovička J., Řanda Z., Jelínek E. (2006). Antimony content of macrofungi from clean and polluted areas. *Chemosphere*.

[20] Liu H. G., Wang Y. Z., Li T. (2012). ICP-AES determination of 11 metals in 16 species of wild mushrooms from Yunnan province. *Chinese Journal of Spectroscopy Laboratory*.

[21] Aloupi M., Koutrotsios G., Koulousaris M., Kalogeropoulos N. (2012). Trace metal contents in wild edible mushrooms growing on serpentine and volcanic soils on the island of Lesvos, Greece. *Ecotoxicology and Environmental Safety*.

[22] Gadd G. M. (2007). Geomycology: biogeochemical transformations of rocks, minerals, metals and radionuclides by fungi, bioweathering and bioremediation. *Mycological Research*.

[23] Zhou L. X., Yin J. Z. (2008). Yunnan wild edible Boletus nutrition analysis and evaluation. *Edible Fungi*.

[24] Li T., Wang Y., Zhang J., Zhao Y., Liu H. (2011). Trace element content of *Boletus tomentipes* mushroom collected from Yunnan, China. *Food Chemistry*.

[25] Liu H. G., Zhang J., Li T., Shi Y., Wang Y. (2012). Mineral element levels in wild edible mushrooms from Yunnan, China. *Biological Trace Element Research*.

[26] Jarzyńska G., Chojnacka A., Dryzalowska A., Nnorom I. C., Falandysz J. (2012). Concentrations and bioconcentration bactors of minerals in Yellow-Cracking Bolete (*Xerocomus Subtomentosus*) mushroom collected in Notec forest, Poland. *Journal of Food Science*.

[27] Kułdo E., Jarzyńska G., Gucia M., Falandysz J. (2014). Mineral constituents of edible parasol mushroom *Macrolepiota procera* (Scop. ex Fr.) Sing and soils beneath its fruiting bodies collected from a rural forest area. *Chemical Papers*.

[28] Gençcelep H., Uzun Y., Tunçtürk Y., Demirel K. (2009). Determination of mineral contents of wild-grown edible mushrooms. *Food Chemistry*.

[29] Jarzyńska G., Falandysz J. (2012). Metallic elements profile of Hazel (Hard) Bolete (*Leccinum griseum*) mushroom and associated upper soil horizon. *African Journal of Biotechnology*.

[30] Sesli E., Tuzen M., Soylak M. (2008). Evaluation of trace metal contents of some wild edible mushrooms from Black sea region, Turkey. *Journal of Hazardous Materials*.

[31] Yamaç M., Yildiz D., Sarikürkcü C., Çelikkollu M., Solak M. H. (2007). Heavy metals in some edible mushrooms from the Central Anatolia, Turkey. *Food Chemistry*.

[32] Zhu P., Guo Y. H., Ding X. W. (2006). Analysis components of 4 kinds of fresh *Boletus* sp. *Edible Fungi of China*.

[33] Mendil D., Uluözlü Ö. D., Hasdemir E., Çağlar A. (2004). Determination of trace elements on some wild edible mushroom samples from Kastamonu, Turkey. *Food Chemistry*.

[34] Zhang D., Frankowska A., Jarzyńska G. (2010). Metals of king bolete (*Boletus edulis*) bull.: Fr. collected at the same site over two years. *African Journal of Agricultural Research*.

[35] Vetter J., Hajdú C., Györfi J., Maszlavér P. (2005). Mineral composition of the cultivated mushrooms *Agaricus bisporus*, *Pleurotus ostreatus* and *Lentinula edodes*. *Acta Alimentaria*.

[36] Vetter J. (2003). Chemical composition of fresh and conserved *Agaricus bisporus* mushroom. *European Food Research and Technology*.

[37] Falandysz J., Kunito T., Kubota R. (2008). Multivariate characterization of elements accumulated in King Bolete *Boletus edulis* mushroom at lowland and high mountain regions. *Journal of Environmental Science and Health Part A*.

[38] Zhang D., Zhang Y., Morawska E. (2013). Trace elements in *Leccinum scabrum* mushrooms and topsoils from Kłodzka Dale in Sudety Mountains, Poland. *Journal of Mountain Science*.

[39] Chen X.-H., Zhou H.-B., Qiu G.-Z. (2009). Analysis of several heavy metals in wild edible mushrooms from regions of China. *Bulletin of Environmental Contamination and Toxicology*.

[40] Wang Z. P., Fan D. Y., Huang Q. H., Zhao Y. Z., Ruan S. Q. (2011). Study on the content of mineral element in several kinds of wild edible fungi and the correlation with the soil. *Journal of Anhui Agriculture*.

[41] Jerzy F., Frankowska A., Jarzyńska G., Anna D., Kojta A. K., Zhang D. (2011). Survey on composition and bioconcentration potential of 12 metallic elements in king bolete (*boletus edulis*) mushroom that emerged at 11 spatially distant sites. *Journal of Environmental Science and Health Part B: Pesticides, Food Contaminants, and Agricultural Wastes*.

[42] Ma P., Zhang D., Yang L. B. (2012). Bioaccumulation of heavy metal in wild edible *Boletus* fruiting body. *Environmental Science & Technology*.

[43] Chudzyński K., Falandysz J. (2008). Multivariate analysis of elements content of Larch Bolete (*Suillus grevillei*) mushroom. *Chemosphere*.

[44] Isildak Ö., Turkekul I., Elmastas M., Aboul-Enein H. Y. (2007). Bioaccumulation of heavy metals in some wild-grown edible mushrooms. *Analytical Letters*.

